# Using ‘infodemics’ to understand public awareness and perception of SARS-CoV-2: A longitudinal analysis of online information about COVID-19 incidence and mortality during a major outbreak in Vietnam, July—September 2020

**DOI:** 10.1371/journal.pone.0266299

**Published:** 2022-04-07

**Authors:** Ha-Linh Quach, Thai Quang Pham, Ngoc-Anh Hoang, Dinh Cong Phung, Viet-Cuong Nguyen, Son Hong Le, Thanh Cong Le, Thu Minh Thi Bui, Dang Hai Le, Anh Duc Dang, Duong Nhu Tran, Nghia Duy Ngu, Florian Vogt, Cong-Khanh Nguyen

**Affiliations:** 1 Department of Communicable Diseases Control, National Institute of Hygiene and Epidemiology, Hanoi, Vietnam; 2 National Centre for Epidemiology and Population Health, Research School of Population Health, College of Health and Medicine, Australian National University, Canberra, ACT, Australia; 3 Department of Biostatistics and Medical Informatics, School of Preventive Medicine and Public Health, Hanoi Medical University, Hanoi, Vietnam; 4 National Agency for Science and Technology Information, Ministry of Science and Technology, Hanoi, Vietnam; 5 HPC SYSTEMS Inc., Tokyo, Japan; 6 CMetric JSC Inc., Hanoi, Vietnam; 7 INFORE Technology Inc., Hanoi, Vietnam; 8 Department of Health Communication and Reward, Ministry of Health, Hanoi, Vietnam; 9 National Institute of Hygiene and Epidemiology, Hanoi, Vietnam; 10 The Kirby Institute, University of New South Wales, Sydney, NSW, Australia; 11 Field Epidemiology Training Program, National Institute of Hygiene and Epidemiology, Hanoi, Vietnam; Chung Shan Medical University, TAIWAN

## Abstract

**Background:**

Trends in the public perception and awareness of COVID-19 over time are poorly understood. We conducted a longitudinal study to analyze characteristics and trends of online information during a major COVID-19 outbreak in Da Nang province, Vietnam in July-August 2020 to understand public awareness and perceptions during an epidemic.

**Methods:**

We collected online information on COVID-19 incidence and mortality from online platforms in Vietnam between 1 July and 15 September, 2020, and assessed their trends over time against the epidemic curve. We explored the associations between engagement, sentiment polarity, and other characteristics of online information with different outbreak phases using Poisson regression and multinomial logistic regression analysis. We assessed the frequency of keywords over time, and conducted a semantic analysis of keywords using word segmentation.

**Results:**

We found a close association between collected online information and the evolution of the COVID-19 situation in Vietnam. Online information generated higher engagements during compared to before the outbreak. There was a close relationship between sentiment polarity and posts’ topics: the emotional tendencies about COVID-19 mortality were significantly more negative, and more neutral or positive about COVID-19 incidence. Online newspaper reported significantly more information in negative or positive sentiment than online forums or social media. Most topics of public concern followed closely the progression of the COVID-19 situation during the outbreak: development of the global pandemic and vaccination; the unfolding outbreak in Vietnam; and the subsiding of the outbreak after two months.

**Conclusion:**

This study shows how online information can reflect a public health threat in real time, and provides important insights about public awareness and perception during different outbreak phases. Our findings can help public health decision makers in Vietnam and other low and middle income countries with high internet penetration rates to design more effective communication strategies during critical phases of an epidemic.

## Introduction

Infodemics, defined as “rapid and far-reaching spread of both accurate and inaccurate information about an emerging event” [1], has emerged as an area of concern during the COVID-19 pandemic [2]. WHO considers infodemics to create ambiguity and distrust between population and government officials, thus mitigate public health policy to prevent and contain the disease [3].

Vietnam implemented a series of public health interventions in combat with COVID-19. During the first half of 2020, Vietnam had successfully contained the outbreak with no COVID-19 related deaths, and a 99-consecutive-day duration without community transmission [4, 5]. On 25 July 2020, a surge of locally acquired COVID-19 cases were identified in Da Nang City in Central Vietnam, a center for foreign trade activities and tourism [6–8]. The outbreak quickly spread to more than 10 provinces and cities across Vietnam, generating nearly 400 cases and causing 35 fatalities in total [9]. This outbreak marked the biggest COVID-19 outbreak in the country during 2020, and also the first with COVID-19 deaths. By the end of August 2020, the outbreak was declared under control. During this time, public awareness and perceptions were of paramount as information of daily COVID-19 situation was broadcasted widely in all types of media [10, 11].

Online platforms can provide rich information to predict and explain the evolution of outbreaks, at the same time be reflective of public awareness and perceptions. Analysis of online data has become a focus area in medical informatics research in recent years [12–14]. Online information was used to research ‘infodemics’ and ‘infodemiology’ for Ebola [15, 16], MERS-CoV [16], and other public health concerns [17]. COVID-19 has also been in the focus of media coverage all over the world, obtaining highest online public attention. Recent research measured behavioral awareness and public attention in responses to COVID-19 using data from popular online media [18–21]. While most of these ‘infodemics’ studies on COVID-19 focused on countries with sustained community transmission, evidence from countries with localized transmission and clusters following case importation is scarce.

With relatively low number of cases and no deaths due to COVID-19 recorded before the Da Nang outbreak, online information about the evolving COVID-19 situation at that time provides a unique opportunity to gain an in-depth understanding of how population engaged and responded online, as well as how online information of COVID-19 were disseminated across platforms. We aimed to analyze characteristics and trends of online information reporting the Da Nang outbreak in order to understand public awareness and perceptions during an unfolding epidemic.

## Materials & methods

### Study design

We collected online information posted on popular online platforms and social media operated in Vietnam between 1 July to 15 September 2020 that focused on the COVID-19 outbreak in Da Nang, Vietnam, in particular about COVID-19 incidence and mortalities. We divided the study period according to the three phases of outbreak in Da Nang: (i) Pre-outbreak (1–24 July 2020); (ii) during the outbreak (25 July– 31 August 2020); (iii) post-outbreak (1–15 September 2020).

### Data collection

Inclusion criteria for online content were: (i) related to COVID-19 incidence or mortalities (identified through pre-defined keywords in S1 Table); (ii) posts were published in ‘public mode’ and remained in the public domain at the time of data collection; (iii) posts were made and posted in the format of posts on social media networks, entries on online forums, and online newspaper contributions; (iv) the geographical area from where the posts were uploaded is Vietnam. Exclusion criteria were: (i) being unrelated to the study topic (i.e. not containing pre-defined keywords in S1 Table); (ii) not being in the public domain at time of collection; and (iii) not generated in Vietnam geographically.

We used the software package “Social Media Command Center” (http://smcc.vn) used by the Vietnam Ministry of Science and Technology for online data collection. This software has been routinely used by National Steering Committee of COVID-19 Prevention in Vietnam since the start of the COVID-19 pandemic to assess public understanding and perception of public health interventions. Data source for collection included public social media networks, popular online forums, and leading online newspapers in Vietnam (S2 Table) [22–25]. Based on a pre-identified keyword search to cover the study topics (S1 Table), we extracted the following data from each included online posts: (i) source, (ii) influence score, (iii) date of posting, (iv) engagement level, (v) sentiment polarity and (vi) content (S3 Table). Influence score was categorized through number of followers and/or views of source of posting (S4 Table), and sentiment polarity was processed and categorize into sentiment based on Vietnamese Lexicon Sentimental Dictionary developed by Tran et al. [26] (S3 Table).

### Data processing

We used the Vietnamese word segmentation package “VnCoreNLP” packages [27] on Python 3.8 to segment words in each post, then processed to delete Vietnamese stop words and clean special symbols.

### Data analysis

We plotted the number of posts and number of COVID-19 incidence and mortality by date to explore awareness and perception with regards to the Da Nang outbreak over time. Variables were summarized by frequency and percentage, and differentiated between the three outbreak periods (before, during, and after the outbreak) by Chi square or Fisher’s exact tests. We summarized the influence score by calculating means and standard deviations (SD). We used the Spearman correlation coefficient to explore the correlation between COVID-19 incidence and mortality reported in Vietnam with the number of posts over time. We used multinomial logistic regression to assess the predictive relationship between sentiment polarity and outbreak periods adjusted for the posts’ variables, reporting odds rations (OR) and 95% confidence intervals (CI). We used zero inflated Poisson regression to explore the relationship between engagement levels and outbreak periods adjusted for the posts’ variables, reporting relative risks (RR), robust standard errors (SE) and 95%CI. These analyses were performed in Stata 16.0.

From the word segmentation, we calculated word frequencies to identify high-frequency keywords stratified by the three outbreak periods using the “NLTK” software package [28]. After extracting the most common words in each topic, we constructed a word-word co-occurrence matrix using “NetworkX” [29] in Python 3.8. We then extracted the matrix to VOSViewer software [30] to create a network of word co-occurrence analysis and cluster analysis, by using the co-occurrence frequency as the edge weight, and word frequency as node weight. We set 100 random run starts and 100 iterations for every optimization algorithm of clustering to run. In the network, the larger the size of the nodes would be, the higher number of links the node would have with its neighbours. The connection between the nodes would indicate that the keywords on the two nodes had appeared together, the stronger the connection would be, the higher the frequency of word co-occurrence and the closer the connection would be between the nodes. Clusters were formed by ranking keywords by both its co-occurrence weight and frequency, meaning keywords that appears both more frequently together and with similar level of frequency were clustered together. Nodes of the same cluster in each network were grouped by colour.

### Ethics statement

This research was approved by the Australian National University’s Human Research Ethics committee (Protocol 2020/605) and the Vietnam National Institute of Hygiene and Epidemiology’s Institutional Review Board (NIHE IRB– 29/2020). We only collected information that was openly available on the internet. Data collection and analysis complied with the terms and conditions for the data sources and the requirements of the respective ethics committees.

## Results

**Fig 1:**
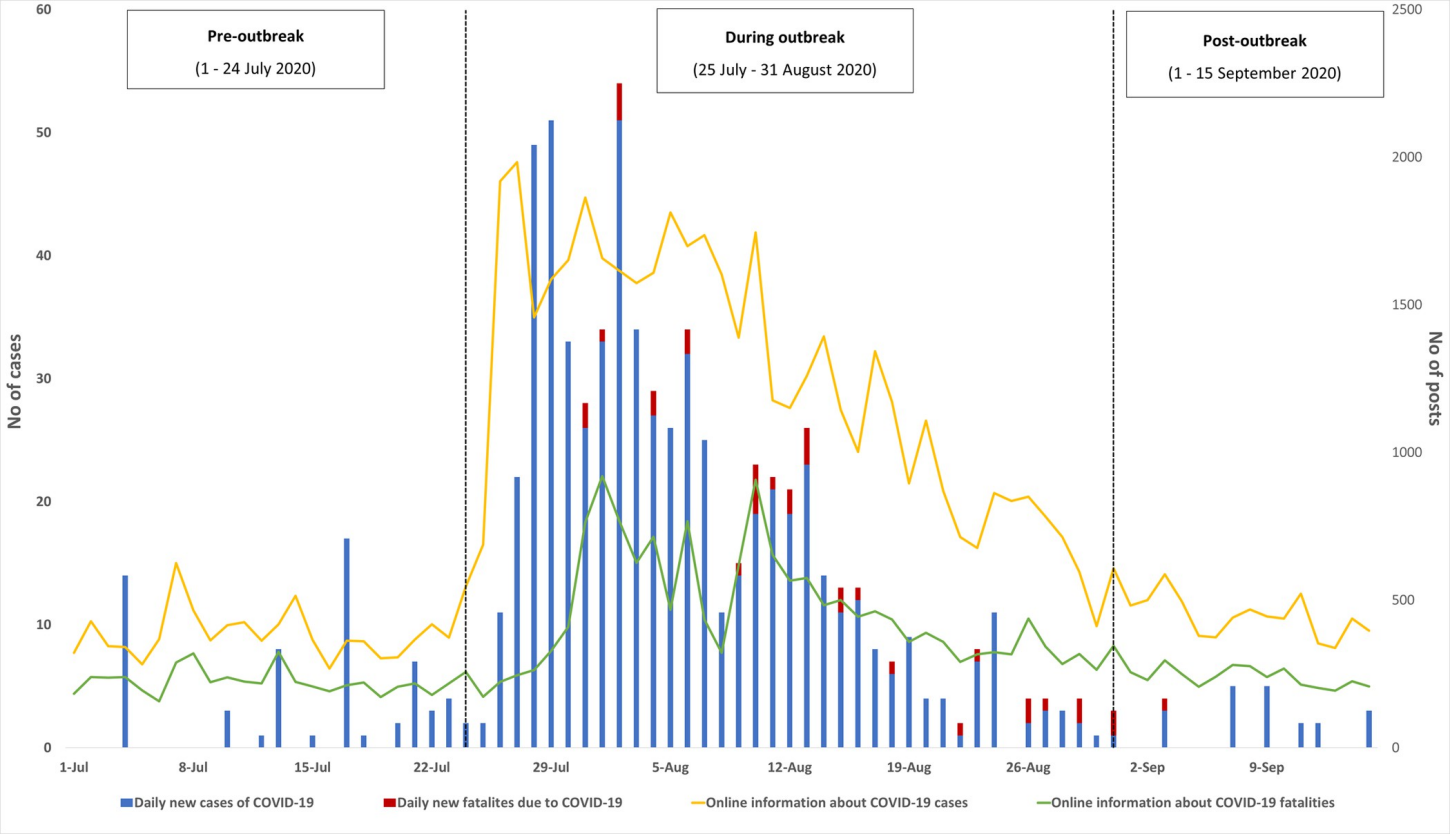
Distribution of online information and number of COVID-19 incidence and mortality in Vietnam divided into three outbreak periods: Pre-outbreak (1–24 July 2020), during outbreak (25 July– 31 August 2020), and post-outbreak (1–15 September 2020). The yellow line indicates daily number of online information about COVID-19 incidence, the green line indicates daily number of online information about COVID-19 mortality. The blue bar indicates daily COVID-19 incidence recorded in Vietnam; the red bar indicates daily COVID-19 mortality recorded in Vietnam.

Table 1 and Fig 1 describe the progression of the COVID-19 outbreak in relation to the amount of online information for the three outbreak phases. For both incidence and mortality, a significantly sharp increase in the number of posts was seen during the outbreak. Higher number of posts per day reporting COVID-19 incidence than reporting COVID-19 mortality was observed, especially during the outbreak. Online newspaper was the main source of COVID-19-related online information throughout the study period. While the information source’s influence score for reporting COVID-19 incidence was not different between outbreak periods, we saw a significant decrease in influence score for COVID-19 mortality towards the end of the outbreak. Information about COVID-19 incidence were mostly reported with neutral tone during the outbreak, and transited to more posts in positive tone after the outbreak. Meanwhile, negative news about COVID-19 mortality were dominant throughout the three periods. Pearson correlation analysis showed both number of posts reporting COVID-19 incidence and COVID-19 mortality was positively correlated with daily incidence of COVID-19 (Pearson coefficient (r) = 0.7852, *P* < .001 and r = 0.6479, *P* < .001, respectively) and daily fatality of COVID-19 (r = 0.4310, *P* < .001 and r = 0.7353, *P* < .001, respectively).

**Table 1:** Description of online information reporting COVID-19 incidence and mortality stratified by outbreak periods.

**A: Incidence**							
**Variables**	**Pre-outbreak**		**During outbreak**		**Post-outbreak**		***P*-value**
	**No. (n)**	**Percentage (%)**	**No. (n)**	**Percentage (%)**	**No. (n)**	**Percentage (%)**	
**Number of posts per day**	389.25		1208.95		443.60		
**Source**							
Social media	2239	23.97	13775	29.22	543	8.16	< .001^a^
Online forum	333	3.56	2190	4.64	136	89.80	
Online newspaper	6770	72.47	31184	66.14	5975	2.04	
**Sentiment polarity**							
Positive	3049	32.64	16348	34.67	2731	41.04	< .001^a^
Neutral	3060	32.76	17597	37.32	1851	27.82	
Negative	3233	34.61	13204	28.00	2072	31.14	
**Influence score (mean, SD)**	4.63 (3.15)		4.64 (3.13)		4.59 (3.14)		.380^b^
**B: Mortality**							
**Variables**	**Pre-outbreak**		**During outbreak**		**Post-outbreak**		***P*-value**
	**No. (n)**	**Percentage (%)**	**No. (n)**	**Percentage (%)**	**No. (n)**	**Percentage (%)**	
**Number of posts per day (mean)**	224.58		512.03		238.87		
**Source**							
Social media	707	13.12	3374	19.38	119	3.32	< .001^a^
Online forum	264	4.90	718	3.12	124	3.46	
Online newspaper	4419	81.99	13317	76.49	3340	93.22	
**Sentiment polarity**							
Positive	1620	30.06	4473	25.69	1202	33.55	< .001^a^
Neutral	1358	25.19	5810	33.37	905	25.26	
Negative	2412	44.75	7126	40.93	1476	41.19	
**Influence score (mean, SD)**	4.88 (3.33)		4.52 (3.25)		3.60 (3.21)		< .001^b^

^a^ *P*-value was calculated by Chi-square test.

^b^ *P*-value was calculated by Fisher’s exact test.

Table 2 shows the sentiment polarity distribution of online information. During the outbreak, neutral information was dominating, while there was more online information with positive and negative sentiment before and after the outbreak. While the majority of social media and online forum posts were made in neutral sentiment, the opposite was true for online newspapers. More positive and neutral posts about COVID-19 incidence were seen, and more negative posts on COVID-19 mortality were observed compared to the other sentiments.

**Table 2:** Distribution of sentiment polarity across posts’ characteristics.

Variables	Positive sentiment (N = 29,423)		Neutral sentiment (N = 30,581)		Negative sentiment (N = 29,523)	
	n	%	n	%	n	%
**Outbreak periods**						
Pre-outbreak	4669	15.87	4418	14.45	5645	19.12
During outbreak	20821	70.76	23407	76.54	20330	68.86
Post-outbreak	3933	13.37	2756	9.01	3548	12.02
**Source**						
Social media	4321	14.69	11875	38.83	4561	15.45
Online forum	467	1.59	2460	8.04	838	2.84
Online newspaper	24635	83.73	16246	53.12	24124	81.71
**Topic**						
Incidence	22128	75.21	22508	73.60	18509	62.69
Mortality	7295	24.79	8073	26.40	11014	37.31

Table 3 shows the multinominal logistic regression analysis of three categories of posts’ sentiment polarity with neutral sentiment as reference category. After adjusting for influence score, sources, and topics, the posts’ sentiment polarity showed a significant association with the outbreak phases, with both information in positive and negative sentiment being less likely to be posted during the outbreak than before and after the outbreak compared to which in neutral sentiment. Online newspapers were also significantly more likely to contain information in negative and positive sentiment than neutral sentiment as compared to social media (OR 4.11 (3.94–4.29), *P* < .001 and OR 3.58 (3.44–3.72), *P* < .001 respectively). Posts’ topics were positively associated with posts’ sentiments, as posts about mortality were more likely to be negative and less likely to be positive than being neutral in posts about incidence (OR 1.43 (1.38–1.48), *P* < .001 and OR 0.77 (0.76–0.82), *P* < .001 respectively).

**Table 3:** Multinominal logistic regression of sentiment polarity over outbreak periods adjusted for posts’ influence score, sources, and topics, using posts in neutral sentiment as reference category.

Variables	Unadjusted analyse		Adjusted analyses	
	Crude OR (95% CI)	*P*-value	Adjusted OR (95% CI)	*P*-value
**A: Posts with positive sentiment** (vs. neutral sentiment)				
**Outbreak periods**				
During outbreak	*Ref*		*Ref*	
Pre-outbreak	1.19 (1.13–1.24)	< .001	1.11 (1.06–1.16)	< .001
Post-outbreak	1.60 (1.52–1.69)	< .001	1.17 (1.11–1.24)	< .001
**Source**				
Social media	*Ref*		*Ref*	
Online forum	0.53 (0.47–0.58)	< .001	0.53 (0.47–0.59)	< .001
Online newspaper	4.17 (4.00–4.34)	< .001	4.11 (3.94–4.29)	< .001
**Topic**				
Incidence	*Ref*		*Ref*	
Mortality	0.92 (0.87–0.95)	< .001	0.77 (0.76–0.82)	< .001
**Influence score**	1.05 (1.04–1.05)	< .001	1.03 (1.02–1.03)	< .001
**B: Posts with negative sentiment (**vs. neutral sentiment)				
**Outbreak periods**				
During outbreak	*Ref*		*Ref*	
Pre-outbreak	1.47 (1.41–1.54)	< .001	1.31 (1.25–1.37)	< .001
Post-outbreak	1.48 (1.41–1.56)	< .001	1.06 (1.00–1.12)	.036
**Source**				
Social media	*Ref*		*Ref*	
Online forum	0.89 (0.81–0.97)	.006	0.85 (0.78–0.92)	< .001
Online newspaper	3.87 (3.72–4.02)	< .001	3.58 (3.44–3.72)	< .001
**Topic**				
Incidence	*Ref*		*Ref*	
Mortality	1.66 (1.60–1.72)	< .001	1.43 (1.38–1.48)	< .001
**Influence score**	1.05 (1.04–1.05)	< .001	1.03 (1.02–1.03)	<0.001

*Note*. Model was calculated by multinomial logistic regression to explore the distribution of positive and negative sentiment polarity over outbreak periods, compared to neutral sentiment polarity, and adjusted for posts’ source, influence score and topics. Model Wald’s likelihood Ratio = 10236.99; *P*-value < .001; Pseudo R2 = 0.0520.

Table 4 shows the distribution of source of information. Across outbreak periods, online newspapers were the main source of information reporting about the COVID-19 situation, both in terms of incidence as well as mortality. Information on social media had higher influence scores (mean 4.84, SD 3.35) than on online forums (mean 4.15, SD 3.08) and online newspapers (mean 4.04, SD 2.45).

**Table 4:** Distribution of source of information across posts’ characteristics.

Variables	Social media (N = 20,757)		Online forum (N = 3,765)		Online newspaper (N = 65,005)		*P*-value
	n	%	n	%	n	%	
**Outbreak periods**							< .001^a^
Pre-outbreak	2,946	14.2	597	15.9	11,189	17.2	
During outbreak	17,149	82.6	2,908	77.2	44,501	68.5	
Post-outbreak	662	3.2	260	6.9	9,315	14.3	
**Sentiment polarity**							< .001^a^
Positive	4,321	20.8	467	12.4	24,635	37.9	
Neutral	11,875	57.2	2,460	65.3	16,246	25.0	
Negative	4,561	22.0	838	22.3	24,124	37.1	
**Topic**							< .001^a^
Incidence	16,557	79.8	2,659	70.6	43,929	67.6	
Mortality	4,200	20.2	1,106	29.4	21,076	32.4	
**Influence score (mean, SD)**	4.84 (3.35)		4.15 (3.08)		4.04 (2.45)		< .001^b^

^a^ *P*-value was calculated by Chi-square test.

^b^ *P*-value was calculated by Fisher’s exact test.

Table 5 presents Poisson regression models for posts’ engagement over outbreak periods. The model adjusted for posts’ source, influence score, sentiment polarity, and topics, showed that collected online information received significantly higher engagement during the outbreak than before or after the outbreak (*P* < .001). Engagement was positively associated with influence score of the source (RR 1.25 (1.24–1.25)), posts reporting COVID-19 mortality in particular had more engagements than posts reporting COVID-19 incidence (RR 1.06 (0.84–1.34)). Posts with neutral sentiment also got significant higher engagements than posts with negative or positive sentiment, while posts on social media received significantly higher engagements than posts on online newspaper and online forum.

**Table 5:** Poisson regression of engagement levels over outbreak periods adjusted for posts’ source, influence score, sentiment polarity, and topics.

Variable	Number of engagements	Unadjusted analysis	Adjusted analyses		
		Crude RR (95%CI)	Adjusted RR (95%CI)	SE	*P*-value
**Outbreak periods**					
During outbreak	11.02×10^6^	*Ref*	*Ref*		
Pre-outbreak	1.05×10^6^	0.58 (0.45–0.75)	0.60 (0.47–0.77)	0.08	< .001
Post-outbreak	0.12×10^6^	0.20 (0.11–0.35)	0.19 (0.11–0.33)	0.05	< .001
**Source**					
Social media	12.1×10^6^	*Ref*	*Ref*		
Online forum	6.95×10^4^	0.08 (0.05–0.12)	0.15 (0.10–0.23)	0.03	< .001
Online newspaper	5.48×10^4^	0.005 (0.005–0.007)	0.001 (0.000–0.0012)	0.00	< .001
**Sentiment polarity**					
Neutral	8.68×10^6^	*Ref*	*Ref*		
Positive	1.69×10^6^	0.42 (0.33–0.54)	0.37 (0.28–0.47)	0.05	< .001
Negative	1.82×10^6^	0.41 (0.32–0.51)	0.37 (0.29–0.47)	0.04	< .001
**Topic**					
Incidence	2.57×10^6^	*Ref*	*Ref*		
Mortality	9.62×10^6^	1.02 (0.81–1.29)	1.06 (0.84–1.34)	0.12	0.605
**Influence score**	--	1.23 (1.22–1.25)	1.25 (1.22–1.27)	0.01	< .001

*Note*. Model was calculated by zero inflated Poisson regression to explore the association between outbreak periods and engagements levels adjusted for posts’ source, influence score, sentiment polarity, and topics. Model Wald’s Likelihood ratio = 180,424.08; *P*-value < .001.

**Fig 2:**
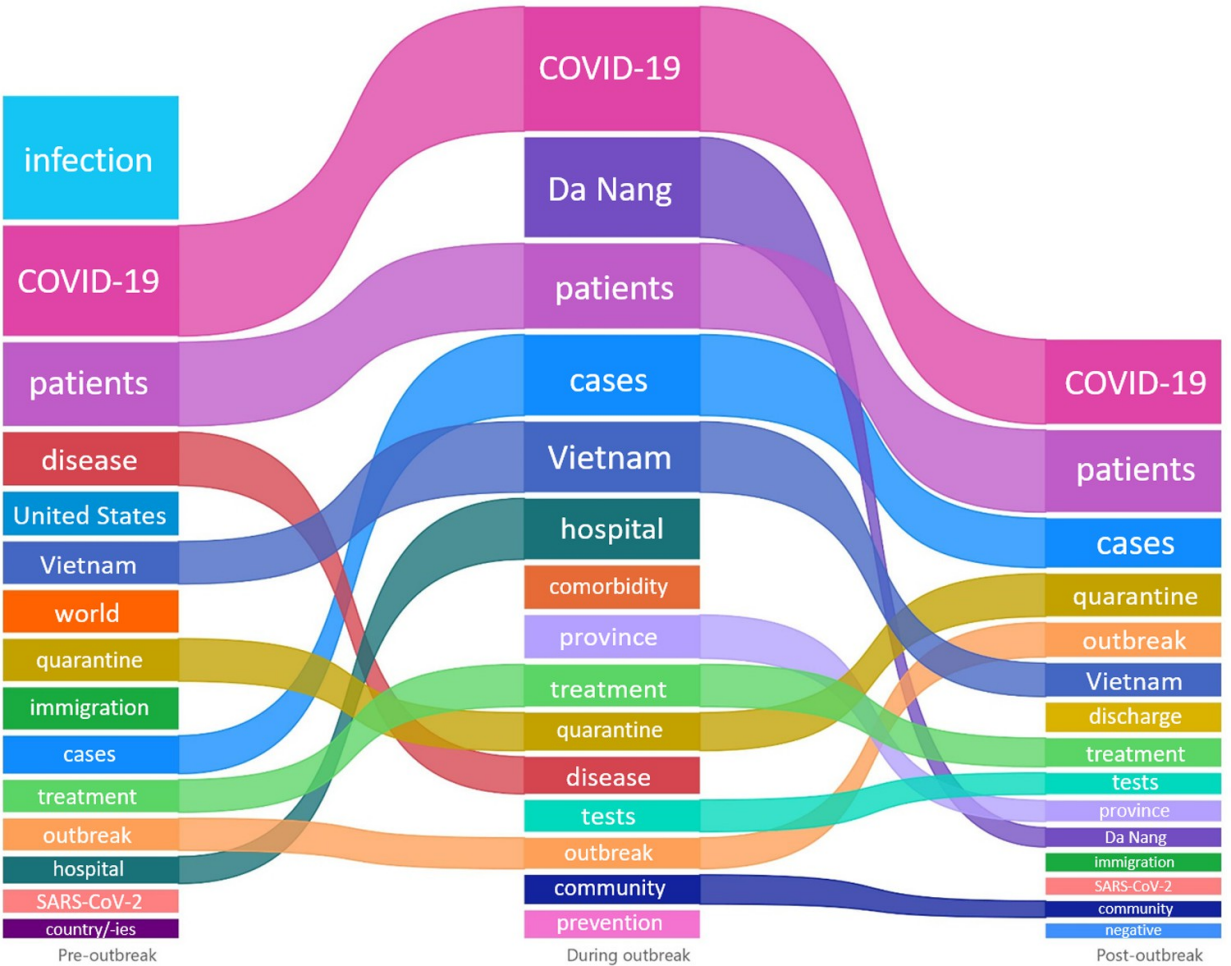
Top 15 keywords with highest appearance frequency in online information about COVID-19 incidence collected divided into three outbreak periods: Pre-outbreak (1–24 July 2020), during outbreak (25 July– 31 August 2020), and post-outbreak (1–15 September 2020).

**Fig 3:**
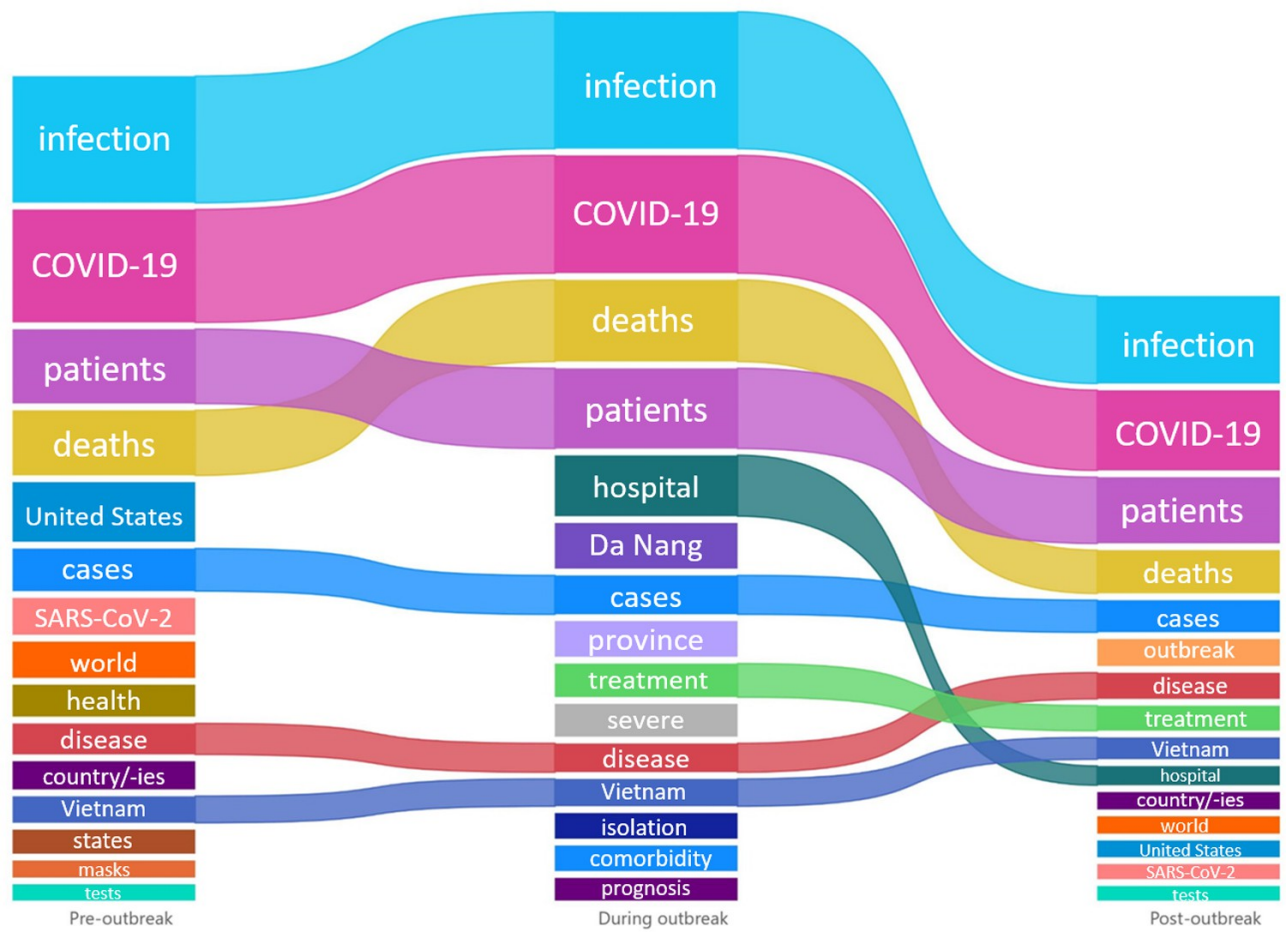
Top 15 keywords with highest appearance frequency in online information about COVID-19 mortality collected divided into three outbreak periods: Pre-outbreak (1–24 July 2020), during outbreak (25 July– 31 August 2020), and post-outbreak (1–15 September 2020).

Figs 2 and 3 show the top 15 frequency words appearing in online posts concerning COVID-19 incidence and mortality, respectively, stratified by the three stages of the outbreak. “COVID-19” and “patients” were the two keywords appearing consistently in all three periods for both topics. Meanwhile, “infection” had the highest frequency in all periods for information reporting COVID-19 mortality, but only in first period for information reporting COVID-19 incidence. Before the outbreak, it showed that COVID-19 situation in the “world”, in particularly in some “states” in “United States”, was covered alongside with Vietnam situation. Meanwhile, no deaths were reported in Vietnam, and all COVID-19 cases in Vietnam at that time were reported cases and were “quarantine” at “immigration”. Compared with the pre-outbreak phase, the during-outbreak phase showed a shift in keywords such as “Da Nang province”, “comorbidity”, “tests”, and “community”. Information about COVID-19 deaths was more articulate, with descriptions of the first COVID-19 deaths reported in Vietnam such as “severe”, “comorbidity” and "prognosis". Into the post-outbreak period, “prevention”, “discharge”, “tests” and “negative” were frequently used keywords. At this stage, the outbreak was under control and more and more cases were "discharged", and the attention had shifted to prevention and control mode in "Da Nang". While the number of new cases remained stagnant for some time, COVID-19 cases with severe prognosis were the main focus of attention in online discussions about COVID-19 deaths. Frequency of each keyword can be found in S5 Table.

**Fig 4:**
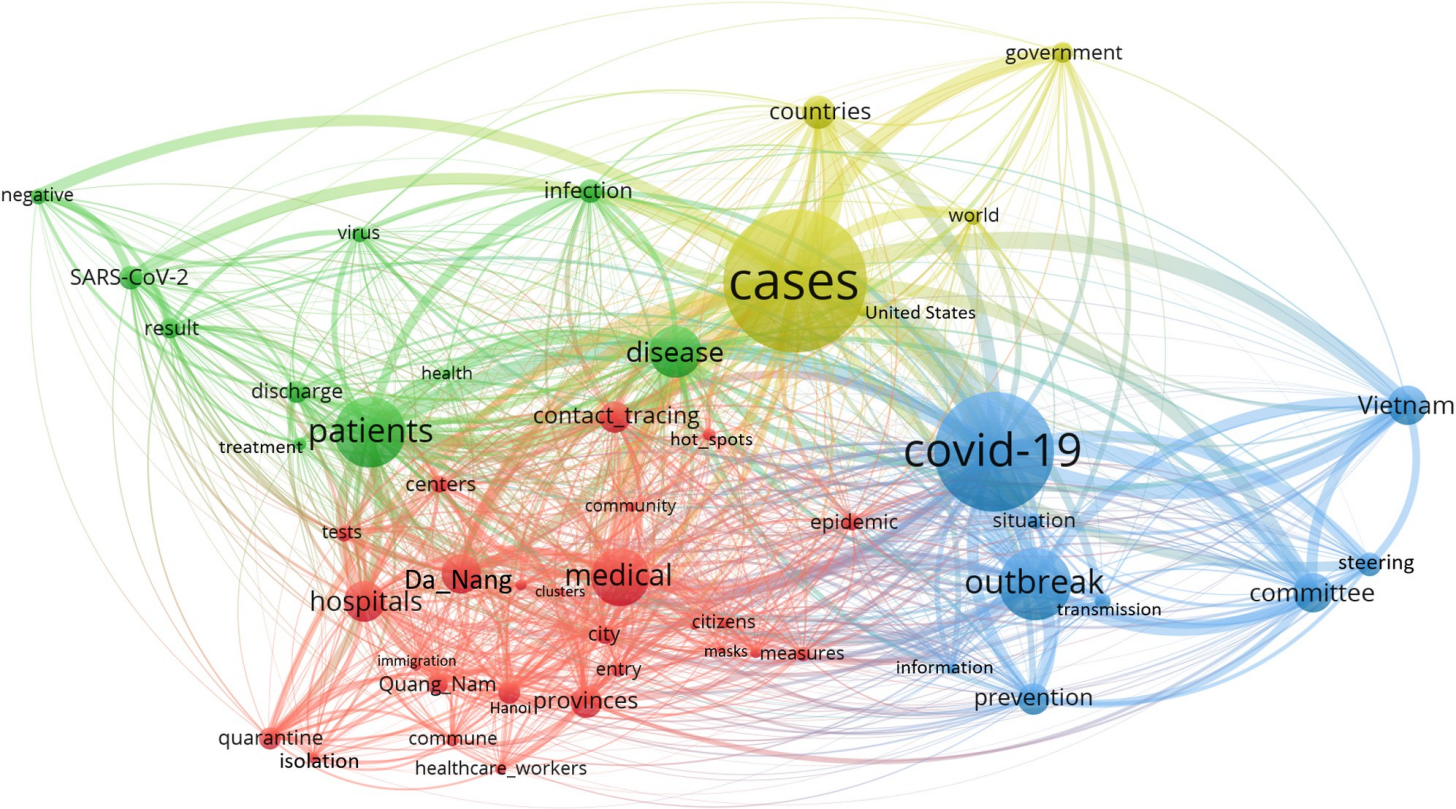
Semantic social network of high-frequency keywords amongst online information about COVID-19 incidence.

**Fig 5:**
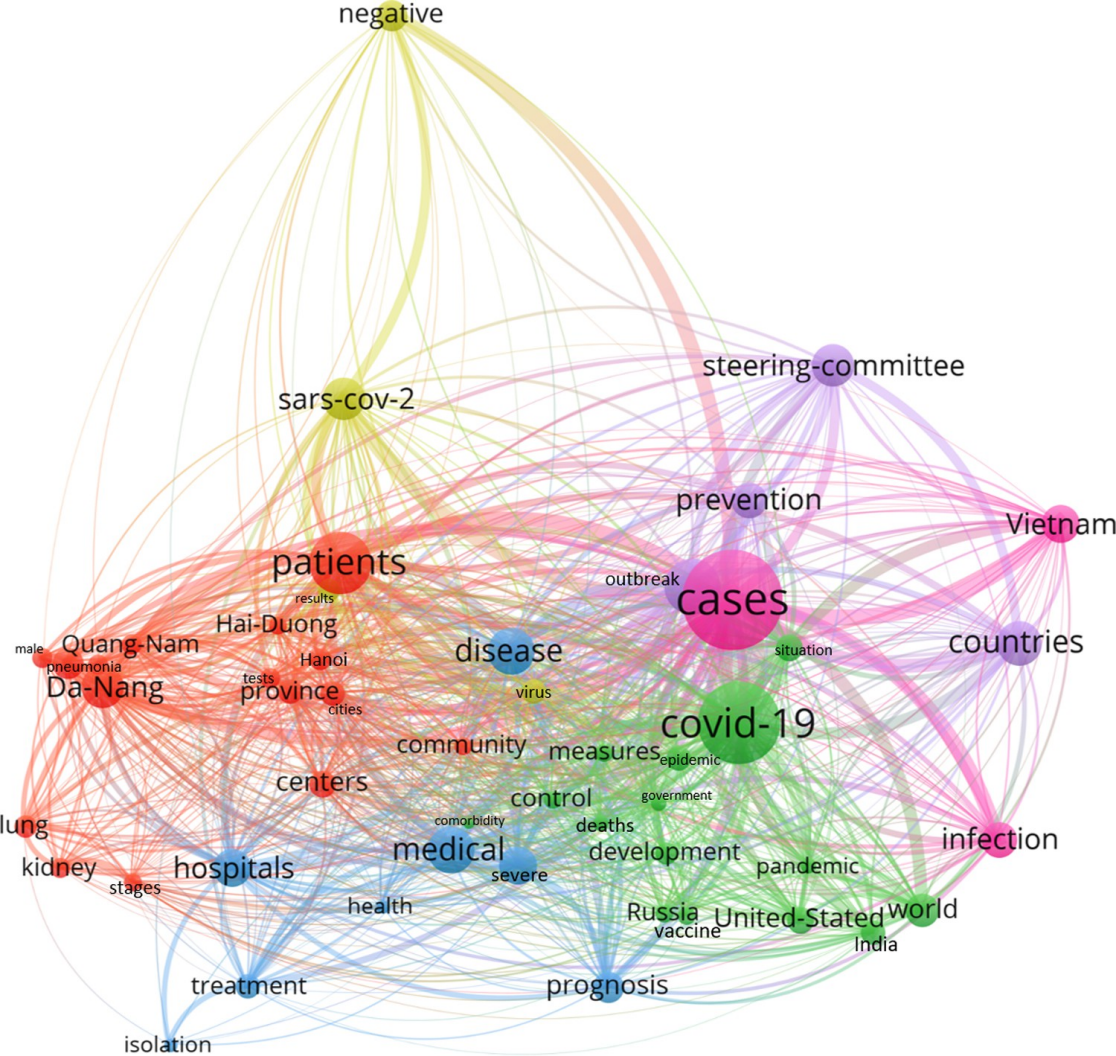
Semantic social network of high-frequency keywords amongst online information about COVID-19 mortality.

Semantic networks of keywords over all three periods are shown in Figs 4 and 5 (Separated networks can be found in S1 and S2 Figs).

“Cases”, “COVID-19”, and “patients” were at core position in the network about COVID-19 incidence (Fig 4) and grouped into four interconnected clusters. Cluster 1 (yellow) involved keywords discussing global COVID-19 situation, including “countries”, “cases”, “United States”, “world”, “government”. Cluster 2 (blue) involved keywords of more domestic view to “Vietnam” National “Steering Committee” of “COVID-19”, and related “outbreak” “transmission” and “prevention” “information”. Cluster 3 (red) was the densest cluster in the network, including keywords reporting situation inside “hot spots” areas: “Da Nang” “city” and “Quang Nam” “province”. The cluster at that time was first detected in local “hospitals”, transmission was rapidly spread to “community” and to other localities including “Hanoi”. Many “measures” were implemented, including extensive “contact tracing”, mass “testing” and “quarantine” “centres”, case “isolation”, “entry” control to “cluster” “commune”, and personal protection such as “masks”. During the outbreak, many “healthcare workers” from other provinces were mobilized to Da Nang for “medical support” to treat COVID-19 cases. Cluster 4 (green) displays information about COVID-19 “patients” “treatment”, which keywords involved “SARS-CoV-2” “virus” testing, “disease”, “infection”, “health”, “negative” “results”, and “discharge”.

Online information of COVID-19 mortality (Fig 5) was grouped into six clusters and had more interconnected nodes between clusters than the network on incidence. “COVID-19”, “cases”, and “patients” were at core of the network with highest links to other keywords. Cluster 1 (purple), cluster 2 (pink), and cluster 3 (yellow) were smaller clusters in the network with only three to four keywords each. While cluster 3 contained information of SARS-CoV-2 testing (featuring “negative”, “result”, “virus”), cluster 1 and 2 presented news about Vietnam’s COVID-19 situation and response (featuring national “Steering committee” of COVID-19 “prevention”, “outbreak”, “country”, and “infection”). Cluster 4 (green) included news about COVID-19 “pandemic” “situation” in “world” view, with “India” and “United States” which had global highest “deaths” count at that time. “Vaccine” “development” in “Russia” was also in the focus, while “governments” were implementing many “control” “measures” for COVID-19. Cluster 5 (blue) involved online information about COVID-19 “treatment”, especially to more “severe” cases at that time. Example of keywords included “hospitals”, “disease”, “health”, “prognosis”, “isolation”, “comorbidity”. Cluster 6 (red) depicts COVID-19 progression in cluster areas–“Da Nang” “City”, “Quang Nam” “province”, and others–“Hanoi” and “Hai Duong”. Since all COVID-19 deaths in Vietnam at that time were cases with pre-existing chronic diseases, keywords such as “lung”, “kidney”, “stages”, and “pneumonia” were in focus.

## Discussion

In this study, we found three strong associations between online information and the evolution of the COVID-19 outbreak. First, three outbreak phases had significant associations with posts’ engagement levels and sentiment polarity. Specifically, online information received significantly higher engagements during the outbreak than before or after the outbreak. Secondly, sentiment polarity was closely associated with posts’ sources, with online newspaper reporting more negative and positive information. There were also significantly more negative posts about COVID-19 mortality and more positive and neutral posts about COVID-19 incidence. Thirdly, keyword analysis and semantic network analysis showed that trending keywords followed closely the evolution of the outbreak.

At the time the Da Nang outbreak started, Vietnam had been virtually COVID-19 free domestically for nearly two months. Unlinked new cases in Da Nang in July [6, 8] certainly alarmed the Vietnamese population. As engagements are highly sensitive and context specific, our findings showed significantly higher engagements during the outbreak than before or after the outbreak. Growing public interest in emerging outbreaks has been explained as reason to engage with online news [16]. Similar trends of online information were observed in early stages of COVID-19 pandemic across the world, of which number of tweets, newspaper, and searches aligned with the increasing COVID-19 incidence [31, 32]. Especially for breaking news such as the first COVID-19 deaths in Vietnam, we observed a significantly higher engagement than for COVID-19 incidence. This was also observed in previous outbreaks of influenza [33], Ebola [34, 35], disaster emergency [17], and now with COVID-19 [36–38].

Despite being the dominant information provider, online newspapers or forums did not receive as many engagements as social media. Similar trends of lower interactions to online forums than mainstream platforms were reported by Cinelli et al. [39]. This can be explained by lower influence score of online newspaper and forums (as in views per article or entries) comparing to that of social media (as in followers per user account), which means online newspaper and forum could not attract as much attention as posts on social media accounts. As Vietnam has repeatedly ranked high in numbers of social media users per capita, and more and more people obtaining news from these platforms [40–42], the impact of social media on perception and awareness on major public events is expected to be more influential than from online newspaper and online forum.

Our sentiment analysis showed an association with the progression of the COVID-19 situation. Previous research also identified similar trends followed by an increase of COVID-19 cases [31, 43]. While no clear impact of sentiments on users’ engagement was observed, neutral information covering the outbreak were dominating our data. As this was not the first community outbreak of COVID-19 in Vietnam, both public and news outlet were more acquainted with the situation. Even though this was the first outbreak after nearly two months, the public was already well aware, and the news was more likely to report the number of cases with neutral-informing tone rather than in emotional sentiment. The same observation was observed in Xu et al., that public opinion was more affected in the beginning, and deeper into the pandemic, sentiment in online news was less polarized [44]. We also saw that negative tone decreased over time as positive tone increased at the end of the outbreak. Similar trends were shown in Yuxin et al. [45] and Sakun et al.’s [46] on COVID-19 posts, as they explained by the close relationship between hazard events, emotions and media [47, 48]. As the epidemic progressed further and eventually got under control, public sentiment tended to skew towards neutral or even positive, as trust in successful responses to the epidemic was strengthened. This was also evident in the use of positive keywords in the latter stage of post-outbreak phase in our research, covering topics of recovering, prevention, and control.

On the other hand, we showed that newspapers were more likely to report information positively or negatively than being neutral, compared to social media and online forum, and most prominently during the outbreak. While newspapers have often been regarded as neutrally reporting sources, the opposite has been observed in the global news coverage of COVID-19 [49–52]. Since COVID-19, newspapers were more likely to portray the pandemic situation from a more negative perspective, especially in heavily-affected countries [31, 43, 52]. Konrad et al. [52] showed a heterogeneity of sentiments in reporting COVID-19 through a substantial volume of negatively-associated newspaper articles, especially in few first month of the pandemic. Both Rizvee et al. [53] and Rao et al. [54] hypothesized that the increasing severity of COVID-19 seen in local context (e.g. in Vietnam, the first COVID-19-related fatalities, the outbreak mongering amongst patients and vulnerable population in hospital) controlled the newspapers, resulted in a majority of warning/negatively-toned news to reframe population perception of the seriousness of the outbreak.

Our findings demonstrate the importance of sources and sentiment polarity in disseminating online information and impact public awareness. Our study approach has implications for future implementation of social media data to public health research and policy. Online data analysis opens new horizons for ‘infodemiology’ and ‘infosurveillance’ for future epidemic. Public health education has realized the power of digital world in facilitating or fabricating information in the quickest and most effective way [56, 57]. Yet, online platforms should not only be used as one-sided information supply tools, but also as an effective multi-way communication channel between the general population and public health agencies. COVID-19 online information spread wider and faster than ever before. Both misinformation and disinformation rely heavily on the uncertainty inherent to concerning situation. This may have also been the reason why we saw higher engagements for online information during the outbreak, when the public was most uncertain about the epidemic progression and how to contain it. From this study, we can see clearly the magnitude, drive, and impact of online information during the unfolding outbreak. It is highly important to recognize influential outlets with higher engagement-driven power, and its impact to polarize (or even distort) public attention and perceptions of the ongoing outbreak. Health agencies should consider utilizing big data tools and analyzing ‘infodemics’ to better understand public reactions and perceptions [58]. Such analyses could show changing levels of public trust and confidence in their country’s public health system, and at the same time help monitor prominent public concerns (both valid or unfounded due to misinformation) about the progression of the epidemic or of public health interventions. At a smaller geographical scale, online information could feed into event-based surveillance tools and thus help public health officials to address misinformation around the epidemic. More importantly, user-generated online data respond very timely to changes in the population’s health needs and information needs, which is invaluable to shape public health messaging and communication strategies.

We acknowledged some limitations of our study. First, our study covered a limited study period, which limits the generalization of the study findings. Secondly, although we did not limit the selection of sources, we did not categorize data source further than newspaper, forum, and social media. While different sources with different authorities and/or reputation would target different audience, our current categorization is relatively broad and unspecific. We also did not collect other information including geospatial distribution or user/followers’ demographics, which would have provided a more comprehensive depiction of online COVID-19 related news. Different sources of online information do not exist independently of each other but have an interactive relationship (for example, many news are shared on the same social media). Hence, similar information can attract different levels of engagement on different platforms. For certain platforms where information must be short and brief (for example Twitter), readers might be more inclined to look at headlines only rather than to click on full text links to online newspaper articles. Yet without newspapers, social media and online forum cannot sustain its audience for important news that require more research and elaboration. More detailed analyses into the interrelationship between sources of information, platforms, and its acclaimed ‘influence’ could be a valuable basis for subsequent research on the drivers and viral ability of online information or ‘infodemics’. Moreover, many posts can report both topics of “incidence” and “mortality”, thus creating overlapping data in the analyses. We could not avoid this overlap entirely in our analysis. Lastly, the concept of online information collectively excluded people with no or poor access to internet. Even though more than 73% of the population in Vietnam has access to the internet in 2021 [59], we could not exclude selection bias of differing awareness and perception of the population segments with poor or no internet access. Therefore, since our study could not capture the general population, extrapolation should not be made carefully to general perceptions of the COVID-19 situation in Vietnam.

## Conclusions

Online information reflected public perceptions toward the epidemic sensitively and timely, both in its coverage and influence. This study was novel in its usage of online data in real-time public health emergencies, and provides a valuable basis to further integrate the strengths of big data analysis of online information into public health research and policy. Our findings can help public health decision makers in Vietnam and other countries with high internet penetration rates to better communicate with population health and information needs, design more effective communication strategies, and translate this into comprehensive prevention and control measures during critical phases of an epidemic.

## Supporting information

S1 Fig Semantic network of keywords appearing in online information concerning COVID-19 incidence in: (A) Pre-outbreak period; (B) During outbreak period; and (C) post-outbreak period.
(TIF)

S2 Fig Semantic network of keywords appearing in online information concerning COVID-19 mortality in: (A) Pre-outbreak period; (B) During outbreak period; and (C) post-outbreak period.
(TIF)

S1 Table Search keywords for online information. (DOCX)

S2 Table Online platforms source for data collection. (DOCX)

S3 Table Definitions of collected variables for online information. (DOCX)

S4 Table Influence score calculation by number of followers and/or views of each source based on built-in function of the SMCC software. (DOCX)

S5 Table Keywords frequency of online information of COVID-19 incidence and mortalities by outbreak periods. (DOCX)
