# Supplementary figures and images for: Using ‘infodemics’ to understand public awareness and perception of SARS-CoV-2: A longitudinal analysis of online information about COVID-19 incidence and mortality during a major outbreak in Vietnam, July—September 2020
Source: PLoS One. 2022 Apr 7;17(4):e0266299. doi: 10.1371/journal.pone.0266299 (PMC8989240; doi:10.1371/journal.pone.0266299)

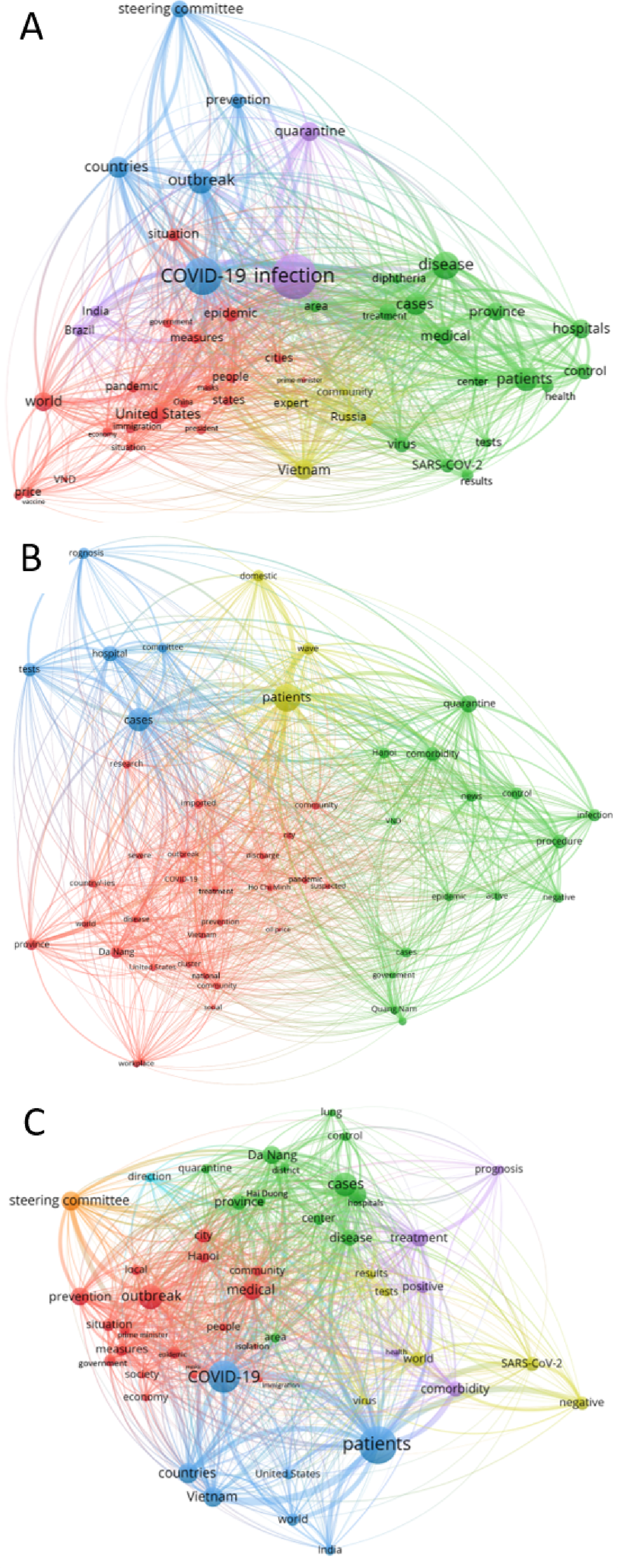

Supplement: S1 Fig — Semantic network of keywords appearing in online information concerning COVID-19 incidence in: (A) Pre-outbreak period; (B) During outbreak period; and (C) post-outbreak period. (TIF) [file pone.0266299.s001.tif]

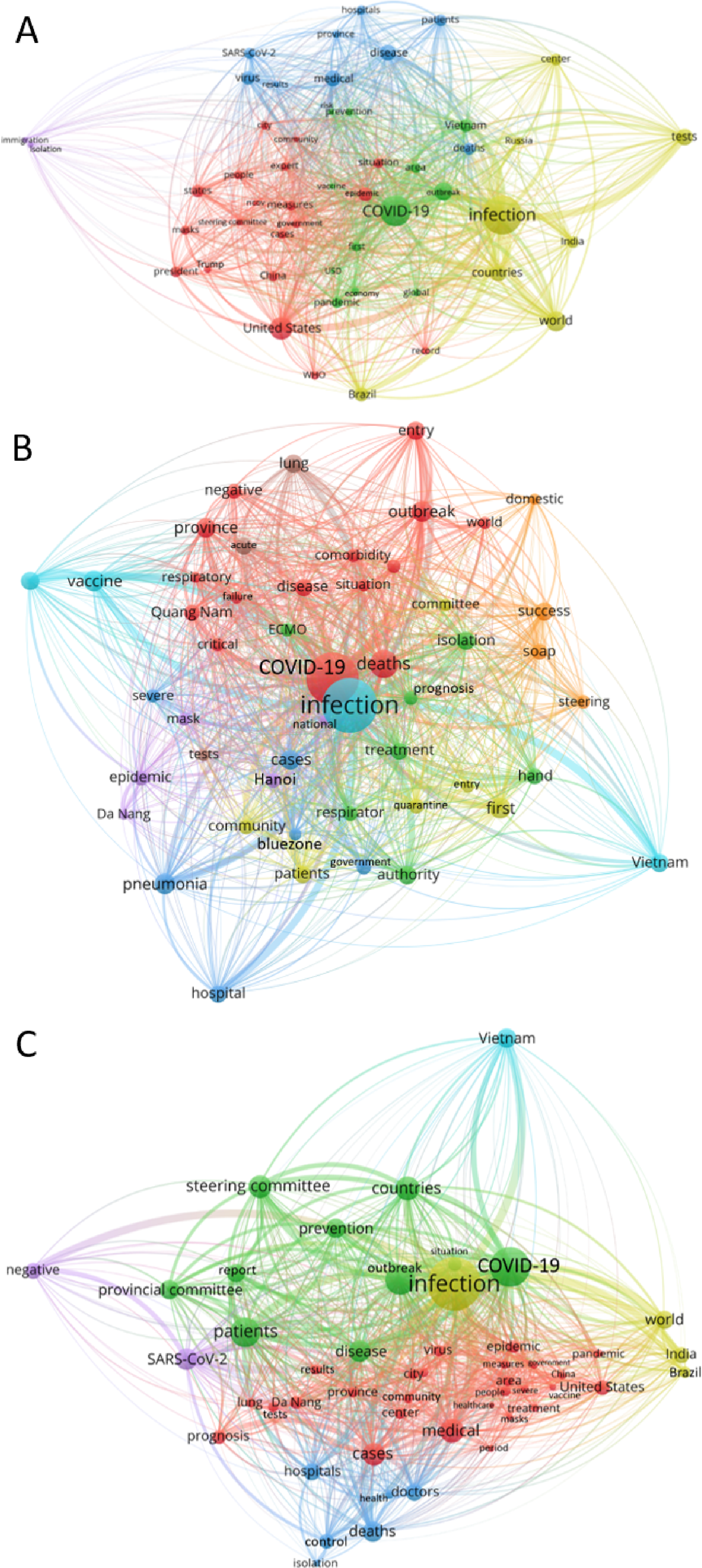

Supplement: S2 Fig — Semantic network of keywords appearing in online information concerning COVID-19 mortality in: (A) Pre-outbreak period; (B) During outbreak period; and (C) post-outbreak period. (TIF) [file pone.0266299.s002.tif]
