# Supplementary material for: Using ‘infodemics’ to understand public awareness and perception of SARS-CoV-2: A longitudinal analysis of online information about COVID-19 incidence and mortality during a major outbreak in Vietnam, July—September 2020
Source: PLoS One. 2022 Apr 7;17(4):e0266299. doi: 10.1371/journal.pone.0266299 (PMC8989240; doi:10.1371/journal.pone.0266299)
Supplement: S1 Table — (DOCX) [file pone.0266299.s003.docx]

| **Topic** | **Search keywords** |
| --- | --- |
| COVID-19 incidence | *Vietnamese*: “ca mắc mới”, “covid”, “dương tính”.  *English:* “new case”, “incidence”, “covid”, “positive” |
| COVID-19 mortalities | *Vietnames*e: “ca tử vong”, “covid”, “t.ử.v.o.n.g”, “t.ử vong”, “c.hết”, “c.h.ế.t.”  *English*: “fatalities”, “mortalities”, “covid”, “deaths”. |
