# Supplementary material for: Using ‘infodemics’ to understand public awareness and perception of SARS-CoV-2: A longitudinal analysis of online information about COVID-19 incidence and mortality during a major outbreak in Vietnam, July—September 2020
Source: PLoS One. 2022 Apr 7;17(4):e0266299. doi: 10.1371/journal.pone.0266299 (PMC8989240; doi:10.1371/journal.pone.0266299)
Supplement: S2 Table — (DOCX) [file pone.0266299.s004.docx]

| **Source** | **Platform name** |
| --- | --- |
| Social media | Facebook, Instagram, Zalo, Zingme, Twitter, etc. |
| Online forums | Tinhte.vn, webtretho.com, lamchame.com, 5giay.vn, vatgia.com, vozforums.com, spiderum.com, chodientu.vn, etc. |
| Online newspapers | dantri.com, vnexpress.com, ngoisao.net, vovnews.vn, nhandan.vn, laodong.vn, etc. |
