# Supplementary material for: Using ‘infodemics’ to understand public awareness and perception of SARS-CoV-2: A longitudinal analysis of online information about COVID-19 incidence and mortality during a major outbreak in Vietnam, July—September 2020
Source: PLoS One. 2022 Apr 7;17(4):e0266299. doi: 10.1371/journal.pone.0266299 (PMC8989240; doi:10.1371/journal.pone.0266299)
Supplement: S3 Table — (DOCX) [file pone.0266299.s005.docx]

| **Variables** | **Retrieving/Categorization method** | **Definition** | **Type** |
| --- | --- | --- | --- |
| Source | Based on the built-in function of the software, each post was labeled by its source from where it was retrieved. | Online platform where the post was initially made public (does not contain identifiable information about who made the post). | Nominal variable:  - Online articles  - Online forum  -Social network |
| Influence score | Based on the built-in function of the software, each source was given a score of influence calculated by number of followers of the source. | Quantitative value of influence that the software calculates for each source of the posts, based on number of followers of the source. | Continuous variable ranking from 1 (lowest influence) to 10 (highest influence) |
| Date of posting | Based on the built-in function of the software. | Date when the post was initially made available on the platform. | Date variable |
| Engagement level | The built-in function of the software retrieved quantitative number of engagements (in form of likes, comments, and shares) of each posts | Quantitative number of engagements (likes, shares, comments) calculated for each post | Discrete variable  - Number of likes  - Number of shares  - Number of comments |
| Sentiment polarity | Based on the built-in function of the software, each posts content was processed through Vietnamese Natural language processing function to identify signal wording and categorize into sentiment based on Vietnamese Lexicon Sentimental Dictionary developed by Tran et al^*^. | Polarity identification of objective textual concept in the posts | Nominal variable  - Negative  - Positive  - Neutral |
| Content | Retrieved by built-in function of SMCC based on selection criteria and selected keywords of the study. | Textual content of the posts that meet the selection criteria and selected keywords of the study (does not contain identifiable information about who made the post). | Text variable |
| ^*^ Tran TK, Phan TT. A hybrid approach for building a Vietnamese sentiment dictionary. J Intell Fuzzy Syst. 2018 Jan 1;35(1):967–78. | | | |
