# Supplementary material for: Using ‘infodemics’ to understand public awareness and perception of SARS-CoV-2: A longitudinal analysis of online information about COVID-19 incidence and mortality during a major outbreak in Vietnam, July—September 2020
Source: PLoS One. 2022 Apr 7;17(4):e0266299. doi: 10.1371/journal.pone.0266299 (PMC8989240; doi:10.1371/journal.pone.0266299)
Supplement: S4 Table — (DOCX) [file pone.0266299.s006.docx]

| **Followers/ Views^*^** | **Influence score** |
| --- | --- |
| Less than 10 | 0 |
| From 10 to 10.000 | 1 |
| From 10.000 to 20.000 | 2 |
| From 20.000 to 50.000 | 3 |
| From 50.000 to 100.000 | 4 |
| From 100.000 to 200.000 | 5 |
| From 200.000 to 500.000 | 6 |
| From 500.000 to 1.000.000 | 7 |
| From 1.000.000 to 2.000.000 | 8 |
| From 2.000.000 to 5.000.000 | 9 |
| From 5.000.000 up | 10 |
| ^*^Views are applied to online newspaper and forum entries, followers are applied for Facebook. | |
