# Supplementary material for: Using ‘infodemics’ to understand public awareness and perception of SARS-CoV-2: A longitudinal analysis of online information about COVID-19 incidence and mortality during a major outbreak in Vietnam, July—September 2020
Source: PLoS One. 2022 Apr 7;17(4):e0266299. doi: 10.1371/journal.pone.0266299 (PMC8989240; doi:10.1371/journal.pone.0266299)
Supplement: S5 Table — (DOCX) [file pone.0266299.s007.docx]

| **Topic: COVID-19 incidence** | | | | | | |
| --- | --- | --- | --- | --- | --- | --- |
| **Rank** | **Pre-outbreak** | | **During outbreak** | | **Post-outbreak** | |
|  | **Keyword** | **Frequency** | **Keyword** | **Frequency** | **Keyword** | **Frequency** |
| 1 | infection | 51315 | COVID-19 | 51671 | COVID-19 | 35005 |
| 2 | COVID-19 | 45861 | Da Nang | 41478 | patients | 34066 |
| 3 | patients | 34738 | patients | 35317 | cases | 20466 |
| 4 | disease | 22181 | cases | 33609 | quarantine | 17706 |
| 5 | United States | 18133 | Vietnam | 29280 | outbreak | 14236 |
| 6 | Vietnam | 17630 | hospital | 25273 | Vietnam | 13871 |
| 7 | world | 17614 | comorbidity | 18005 | discharge | 12140 |
| 8 | quarantine | 17602 | province | 17965 | treatment | 11883 |
| 9 | immigration | 17476 | treatment | 17459 | tests | 8811 |
| 10 | cases | 15756 | quarantine | 15744 | province | 8699 |
| 11 | treatment | 13435 | disease | 15367 | Da Nang | 8026 |
| 12 | outbreak | 13297 | tests | 13138 | immigration | 7616 |
| 13 | hospital | 11179 | outbreak | 12785 | SARS-CoV-2 | 7132 |
| 14 | SARS-CoV-2 | 9548 | community | 12098 | community | 6731 |
| 15 | country/ies | 8012 | prevention | 11621 | negative | 6121 |
| **Topic: COVID-19 mortalities** | | | | | | |
| **Rank** | **Pre-outbreak** | | **During outbreak** | | **Post-outbreak** | |
|  | **Keyword** | **Frequency** | **Keyword** | **Frequency** | **Keyword** | **Frequency** |
| 1 | infection | 37545 | infection | 40560 | infection | 25962 |
| 2 | COVID-19 | 33504 | COVID-19 | 34939 | COVID-19 | 23770 |
| 3 | patients | 22048 | deaths | 24209 | patients | 19640 |
| 4 | deaths | 19302 | patients | 23770 | deaths | 12812 |
| 5 | United States | 17666 | hospital | 18056 | cases | 9494 |
| 6 | cases | 12617 | Da Nang | 13560 | outbreak | 7984 |
| 7 | SARS-CoV-2 | 10959 | cases | 11512 | disease | 7748 |
| 8 | world | 10740 | province | 10583 | treatment | 7398 |
| 9 | health | 9482 | treatment | 9939 | Vietnam | 6463 |
| 10 | disease | 9172 | severe | 9692 | hospital | 5672 |
| 11 | country/ies | 8363 | disease | 8533 | country/ies | 5192 |
| 12 | Vietnam | 7865 | Vietnam | 8084 | world | 5118 |
| 13 | states | 7171 | isolation | 7614 | United States | 4969 |
| 14 | masks | 5030 | comorbidity | 7555 | SARS-CoV-2 | 4505 |
| 15 | tests | 4818 | prognosis | 6748 | tests | 4323 |
